# An *Artemisia ordosica* extract: Effects on growth performance, immune, and inflammatory response in lipopolysaccharide-challenged broilers

**DOI:** 10.3389/fvets.2022.980690

**Published:** 2022-09-09

**Authors:** Lulu Shi, Yanfei Guo, Yuchen Cheng, Yuanyuan Xing, Shiwei Guo, Linghui Zhang, Yuanqing Xu, Xiao Jin, Sumei Yan, Binlin Shi

**Affiliations:** College of Animal Science, Inner Mongolia Agricultural University, Hohhot, China

**Keywords:** *Artemisia ordosica* alcohol extract, broiler, lipopolysaccharide challenge, growth performance, immunomodulation

## Abstract

*Artemisia ordosica* has been applied as a traditional Chinese/Mongolian medicine for treating csertain inflammatory ailments. This study was conducted to investigate the effect of *Artemisia ordosica* alcohol extract (AOAE) supplemented in diets on growth performance, immune, and inflammatory response in lipopolysaccharide (LPS)-challenged broilers. A total of 240 one-day-old Arbor Acre male broilers were randomly allotted into 5 groups with 6 replicates (*n* = 8), which were basal diet group (CON), LPS-challenge and basal diet group (LPS), LPS-challenge and the basal diet added with low (500 mg/kg), middle (750 mg/kg), and high (1,000 mg/kg) dose of AOAE groups (AOAE-L, AOAE-M, and AOAE-H), respectively. On d 16, 18, 20, 22, 24, 26, and 28, all broilers were injected intra-abdominally either with LPS or an equivalent amount of saline. Results showed that dietary AOAE alleviated the LPS-induced decrease in average daily gain and average daily feed intake in the broilers (*P* < 0.05). Dietary AOAE supplementation reversed the increased spleen index and the decreased bursa index in LPS-challenged broilers (*P* < 0.05). Moreover, feeding AOAE could mitigate the elevation of IL-1β in serum, liver, and spleen, IL-2 in serum and liver, IL-6 in serum and spleen, and the decrease of IgG in spleen, IgM in serum, liver, and spleen, and IL-4 in serum of the LPS-challenged broilers (*P* < 0.05). This study also showed that AOAE supplementation alleviated the increase of mRNA expression of *TLR4, MyD88, TRAF6, NF-*κ*B p65, NF-*κ*B p50, IL-1*β, and *IL-6*, and the decrease of gene expression of *I*κ*B*α and *PPAR*γ in liver and/or spleen of broilers challenged by LPS (*P* < 0.05). We speculated that AOAE administration could effectively alleviate LPS-induced inflammation via decreasing over-production of proinflammatory cytokines, ultimately relieving the growth inhibition of broilers caused by LPS. In conclusion, 1,000 mg/kg AOAE has a strong capacity to enhance immunity and inhibit inflammation, and can be used as a potential novel feed additive with applications in treating inflammation-related diseases and bacterial infection in broilers.

## Highlights

- 1,000 mg/kg AOAE supplementation alleviated LPS-induced inflammation in broilers.
- 1,000 mg/kg AOAE supplementation relieved LPS-induced growth inhibition of broilers.
- The results of this study provided a theoretical basis for further developing *Artemisia ordosica* and its extract including AOAE as a feed additive in poultry diets to improve growth performance and immune function.

## Introduction

Over the past decades, the modern broiler farming business has grown rapidly because of the advantages of poultry meat, including high production efficiency, low cost, no religious restriction, and multiple consumption options (1). Referring to related data and reports, the global demand for poultry meat is likely to grow at a peak rate of 121% between 2005 and 2050 (2). Admittedly, broilers can reach market weight in a shorter period of time due to genetic selection, while providing more meat with a higher feed efficiency than ever before via intensive farming model. However, in reality, as intensive breeding has continued to grow, the stress response caused by high farming density has followed, especially birds are more susceptible to a variety of stressors (such as nutritional, physiological/pathological and environmental) than other domestic species. Oxidative stress and inflammatory response are two of the physiological consequences of such stressors in broilers, which have an intense link and influence each other (3). This situation may not only affect broiler welfare but also have adverse effects on antioxidant status and immunity and increase the risks of facing biological damage, disease threats and even mortality, which ultimately result in poor performance and economic losses. According to statistics, the average annual economic burden induced by the prevalence of disease-causing pathogens and their metabolites pose in the global poultry industry is $3 to $6 billion (4). Consequently, with the rapid growth of the broiler farming industry and the fact that broilers continuously suffer from some type of stress, it is extremely urgent to supplement an immune modulator to enhance immune function, thus mitigating the adverse effects of stressors.

Antibiotics have been used as feed additives since 1950s to prevent and control diseases and indirectly accelerate the growth of animals, as well as have made a huge contribution to the poultry industry (5). Nonetheless, it is worth noting that the abuse of antibiotics not only affects the safety of animal products but also induces the emergence of a number of antibiotic resistant human pathogens and causes the deterioration of the ecological environment (6). What's more, following the rise of customers' attention to health and safety issues, the modern livestock production system has also begun to more consider the concept of clean, green, and ethical animal production practices (7). Considering these facts, the prohibition of supplementing antibiotics in diets of livestock have been implemented in many countries of the European Union since 2006, and China has instituted a comprehensive restriction on the use of antibiotics in poultry since 2020 as well (8, 9). Thus, it is imperative to study and develop green, economic, and effective alternatives to antibiotic used in poultry production. Recently, there is increasing interest in natural products, particular to traditional medicinal plants, as a source of alternative to antibiotics in feeds. It has been proven that many of the plant secondary metabolites can modulate the immune responses and disrupt the proinflammatory cascade through antioxidant mechanisms and/or variations in cell signaling, and ultimately, enhance the health of animals and their resistance to pathogens (10). Therefore, the form of herbal extract alternatives is widely applied to protect animals to relieve stress and improve health and performance in the Western as well as in many Asian countries, including China and India (10).

The immune and inflammatory reactions of broilers stimulated by stressors are the main pathogenesis of various immune-inflammatory diseases. Therefore, alleviating inflammation might be an efficient approach to treat inflammation-related diseases. Medicinal plants and their extracts have long been applied in traditional Chinese medicine and have shown potent pharmacological effects on metabolic and inflammatory diseases. *Artemisia ordosica* alcohol extract (AOAE) is isolated from *Artemisia ordosica* Krasch (*A. ordosica, Compositae* family, *Artemisia* genus), which is a traditional Chinese/Mongolian medicine. *A. ordosica* is a main and representative plant in dry areas of East Asia, especially in the north and northwest of China, such as Inner Mongolia, Xinjiang, and the rest (11). The whole plant of *A. ordosica*, even root, has been utilized as a folk medicine to treat rheumatoid arthritis, cold headache, sore throat, carbuncle, swollen boil, and nasal bleeding, etc. (12). In addition, owing to high nutritional value and also rich in flavonoid (13), terpenoids (14), polysaccharides (15), sterols (16), coumarins (17), acetylenes (18), and other bioactive compositions, mainly flavonoid and terpenoid compounds (19–23), *A. ordosica* and their extracts have multiple pharmacological activities, including antimicrobial (24), antioxidation (25), anti-inflammation (26), immunomodulation (27), and therefore can be efficiently applied in poultry and animal production to promote health. Our previous studies have verified that *A. ordosica* aqueous extracts, mainly comprised of flavonoids, terpenoids, organic acids, and polysaccharides, could effectively improve the growth and antioxidant capacity and modulate the immune function of broilers and weanling piglets (26, 27). However, as we have explored, in comparison with ethanol extraction technique, water extraction is lower in extraction rate and the contents of effective composition in extract, while the impurity components are higher. Consequently, as reported by Ghalia (28), an ethanol extract usually shows greater potential for improvement as compared to aqueous extract, which may be attributed to that the ethanolic extracts contain higher concentrations of alkaloids, phenols, steroids, tannins, flavonoids, or glycosides (29).

The endotoxin lipopolysaccharide (LPS), a primary component of the cell wall of gram-negative bacteria, can be recognized by immune cells as a pathogen-associated molecular pattern and consequently induces an inflammatory response, which reduces the feed intake and body weight gain, resulting in the animal's growth being inhibited (30). Intraperitoneal or intravenous injection of LPS can be used to effectively model oxidative stress and inflammatory damage caused by bacterial infection. Our previous study also successfully built inflammatory immune response model of broilers by injecting LPS, meanwhile, verified the *A. ordosica* aqueous extract in the basal diet could effectively alleviate LPS-induced immune overresponse in broilers by lessening the inflammatory cytokines and stress hormone (26). However, whether *A. ordosica* alcohol extract (AOAE) exerts beneficial effects on growth performance and immune function in broilers still remains unclear.

Therefore, the present study used ethanol, which is much less toxic than methanol, as a safer solvent to extract the bioactive components of *A. ordosica*, and aimed to investigate whether AOAE in diets could alleviate inflammatory damage and growth inhibition of broilers induced by LPS, so as to provide a theoretical basis for the application of *A. ordosica* and its alcohol extract in poultry production.

## Materials and methods

This study was conducted after the approval by the Animal Care and Use Committee of Inner Mongolia Agricultural University and performed following the national standard Guideline for Ethical Review of Animal Welfare (GB/T 35892-2018).

### Preparation of AOAE

Fresh *A. ordosica* (aerial part) was collected from Erdos (40.41° N and 110.03° E, Inner Mongolia, China) in July. Raw materials were washed with distilled water and shade-dried at room temperature. The dried materials were smashed and sieved (60 mesh), then the powder was degreased and removed pigments by petroleum ether in the Soxhlet apparatus for 12 h and dried again at room temperature to reduce petroleum ether residues. AOAE was prepared using the method described by Guo et al. (31). Briefly, after a series of above-mentioned processing, 30 g dry powder was steeped in 900 mL 60% ethanol aqueous solution (solid: ethanol = 1: 30), ultrasonic-assisted extraction for 1 h at 200 W power, heated reflux at 50°C to obtain the extracting solution. The resulting solution was filtered through a 0.45 μm filter and the filtrate was concentrated using a rotary vacuum evaporator (RE-5298, Shanghai Yarong Biochemical Instrument Factory, Shanghai, China), and then lyophilized by a vacuum evaporate to prepare the powder which was stored at −20°C until use. The total flavonoid content was determined according to Wang et al. (32), using rutin as a standard. The phenol sulfuric acid method was applied to evaluate total polysaccharide content based on Di et al. (33), and D-glucose was used as a reference standard. Results were expressed as rutin equivalents (mg RE/g) for total flavonoid, D-glucose equivalents (mg GE/g) for total polysaccharide, and the content of total flavonoid and total polysaccharide in AOAE was 556.1 mg RE/g and 145.9 mg GE/g, respectively.

### Birds, experimental design, and diets

A total of 240 similar-sized (38.14 ± 0.36 g) healthy one-day-old Arbor Acres male broilers were purchased from a local commercial hatchery in Hohhot, Inner Mongolia, China, and were randomly divided into five treatment groups with six replicates for each group and eight broilers in each replicate. Using a completely randomized trial design, the five treatments were as follows: (1) control group (CON), broilers received a basal diet and treated with 0.9% sterile saline; (2) LPS group (LPS), broilers received a basal diet and underwent LPS-challenge; (3) low-dose of AOAE group (AOAE-L), broilers received a basal diet supplemented with 500 mg/kg AOAE and underwent LPS-challenge; (4) middle-dose of AOAE group (AOAE-M), broilers received a basal diet supplemented with 750 mg/kg AOAE and underwent LPS-challenge; (5) high-dose of AOAE group (AOAE-H), broilers received a basal diet supplemented with 1,000 mg/kg AOAE and underwent LPS-challenge. According to our previous study, when AOAE level in basal diet was 750 mg/kg, broilers under normal rearing conditions had better growth performance, immunity and antioxidant capacity, therefore, the diet containing 750 mg/kg AOAE was chosen in the current experiment as a medium dosage treatment group. The experiment lasted for 42 days, divided into the starter phase (d 1 to 15), stress period (d 16 to 28) and convalescence (d 29 to 42). During stress period (on d 16, 18, 20, 22, 24, 26 and 28), the broilers were injected intra-peritoneally either with LPS solution (*Escherichia coli*, serotype O55: B5, L2880; Sigma-Aldrich, St. Louis, MO, USA) at the dose of 750 μg/kg of body weight (BW) (LPS was dissolved in sterile saline at a concentration of 100 μg/mL) or with an equal dose of 0.9% sterile saline.

The feeding trial was conducted on the experimental farm of Inner Mongolia Agricultural University, Hohhot, China. According to the method reported by De Oliveira and Lara (34), the incremental lighting system was adopted in the whole experimental period. The temperature of the experimental room was set at 33°C for the first 3 days and then gradually reduced by 3°C every week, and reached a final temperature of 21°C. The relative humidity and ventilation were maintained at about 50–60% and 0.2–0.5 m/s, respectively. All broilers were routinely immunized and had *ad libitum* consumption of diet and water throughout the trial. The vaccination procedure was conducted as follows: the broilers were vaccinated with Newcastle disease and infectious bronchitis combined vaccine on d 7 and 20, Newcastle disease, infectious bronchitis and avian influenza triple vaccine on d 10, infectious bursal disease vaccine on d 14 and 24. All diets were fed in mash form and were based on corn-soybean meal and were formulated to meet or slightly exceed National Research Council (35) recommendations and nutrients recommendations of Feeding Standard of Chicken, China (NY/T 33-2004) (36) (Table 1).

**Table 1:** Composition and nutrient levels of the basal diet (as-fed basis), %.

**Items**	**1 to 21 days of age**	**22 to 42 days of age**
**Ingredients**		
Corn	52.50	58.80
Soybean meal	40.00	33.80
Soybean oil	3.00	3.00
Dicalcium phosphate	1.90	1.80
Limestone	1.08	1.22
Salt	0.37	0.37
Lysine	0.05	0.03
Methionine	0.19	0.07
Premix^1)^	0.80	0.80
Choline chloride	0.11	0.11
Total	100.0	100.0
Nutrient levels^2)^		
Metabolic energy (MJ/kg)	12.42	12.62
Crude protein	21.77	19.65
Calcium	1.00	1.02
Available phosphorus	0.44	0.42
Lysine	1.34	1.15
Methionine	0.55	0.40
Cystine	0.40	0.36

1) Premix provided the following per kilogram of diet: vitamin A 9000 IU, vitamin D_3_ 3000 IU, vitamin E 26 mg, vitamin K_3_ 1.20 mg, vitamin B_1_ 3.00 mg, vitamin B_2_ 8.00 mg, vitamin B_6_ 4.40 mg, vitamin B_12_ 0.012 mg, nicotinic acid 45 mg, folic acid 0.75 mg, biotin 0.20 mg, calcium pantothenate 15 mg, Fe 100 mg, Cu 10 mg, Zn 108 mg, Mn 120 mg, I 1.5 mg, Se 0.35 mg.

2) Crude protein was measured value, while others were all calculated values.

### Sample collection and preparation

Broilers were weighed on days 1, 15, 28, 42, and the feed consumption on each replicate basis was recorded on days 15, 28, and 42 to calculate the average daily gain (ADG), average daily feed intake (ADFI) and feed-to-gain ratio (F: G) for each period.

On d 28 and 42, one broiler was randomly selected from each replicate to collect blood sample from the wing vein using a vacuum non-anticoagulant tube after anesthesia with sodium pentobarbital, and then euthanized by cervical dislocation and dissected to collect the spleen, thymus, bursa of Fabricius and to weigh them after stripping fat. Serum sample was detached and collected via centrifugation at 3,000 × g for 15 min at 4°C and then immediately stored at −20°C until further analysis. The values for immune organ index were calculated by the following equation:


$$\begin{array}{l} Organ\;index=organ\;weight\;\left[g\right]/BW\;\left[kg\right] \end{array}$$

Spleen and liver samples were collected according to the above methods and frozen immediately in liquid nitrogen, then stored at −80°C for preparation of homogenate and total RNA isolation.

### Determination of immune indexes in serum and tissue samples

Liver and spleen tissue samples were minced and homogenized with ice-cold saline (wt/vol, 1: 9), then centrifuged at 4,000 × g for 15 min at 4°C. Interleukin-1 beta (IL-1β), interleukin-2 (IL-2), interleukin-4 (IL-4), interleukin-6 (IL-6), immunoglobulin A (IgA), immunoglobulin G (IgG), and immunoglobulin M (IgM) concentrations in the serum and tissue homogenate supernatant were analyzed using ELISA kits (Quanzhou Ruixin Biological Technology Co., Ltd. Fujian, China) following the manufacturer's instructions. Coomassie brilliant blue assay was used to determine the protein of the homogenate according to the instructions of the commercial kits (Nanjing Jiancheng Institute of Bioengineering, Nanjing, China).

### Total RNA extraction and reverse transcription

Total RNA from liver and spleen samples was obtained using Trizol reagent (TaKaRa Biotechnology Co. Ltd, Dalian, China). The whole extraction process was carried out in a closed sterile environment without enzymes to avoid contamination. The purity and quantity of the total RNA were assessed with a spectrophotometer (Pultton P200CM, San Jose, CA, USA). Subsequently, the total RNA was treated with DNase I (TaKaRa) to remove DNA.

Total RNA was reverse transcribed to cDNA on LifeECO (TC-96/G/H(b)C, BIOER, Hangzhou, China) using TB^®^ Green qPCR method with a Prime Script™ RT reagent kit with gDNA Eraser (TaKaRa Biotechnology Co. Ltd., Dalian, China). The reactions were incubated for 15 min at 37°C, followed by 5 s at 85°C.

### Quantitative Real-time PCR

Real-time PCR was performed using the QuantStudio^®^5 real-time PCR Design & Analysis system (LightCycler^®^ 480 II, Roche Diagnostics, USA) with a TB^®^ Premix Ex Taq™ Kit (Takara Biotechnology Co. Ltd., Dalian, China). The reactions were: 95°C for 30 s (hold stage), followed by 40 cycles of 95°C for 5 s, 60°C for 30 s, and 72°C for 20 s (PCR stage), then 95°C for 15 s, 60°C for 1 min, 95°C for 15 s (melt-curve stage). A subsequent dissociation stage produced a melting curve to verify the specificity of the amplified products as described in the previous report (37). The mRNA expression of each gene was normalized to that of β-actin. The fold change relative to the control group was analyzed according to the 2^−ΔΔCT^ method. The specific sequences of primers are listed in Table 2.

**Table 2:** Primer sequences and parameter.

**Genes**	**GenBank accession No**	**Primer sequences, 5^′^-3^′^**	**Length, bp**	**References**
*TLR4*	NM_001030693	F-TTCAGAACGGACTCTTGAGTGG	131	Xing et al. (15)
		R-CAACCGAATAGTGGTGACGTTG		
*MyD88*	NM_001030962	F-CCTGGCTGTGCCTTCGGA	198	Xing et al. (15)
		R-TCACCAAGTGCTGGATGCTA		
*TRAF6*	XM_421089	F-GAGTGTCCAAGGCGTCAAGTCTG	243	Lu et al. (38)
		R-GTGTCGTGCCAGTTCATTCCTC		
*NF-κB p65*	D13721	F-CAGCCCATCTATGACAACCG	151	Xing et al. (15)
		R-CAGCCCAGAAACGAACCTC		
*NF-κB p50*	NM_205134	F-GAAGGAATCGTACCGGGAACA	80	Cheng et al. (39)
		R-CTCAGAGGGCCTTGTGACAGTAA		
*IκBα*	NM_001001472.2	F-GGCAGATGTGAACAAGGTGA	118	Yang et al. (40)
		R-TATCTGCAGGTCAGCTGTGG		
*IL-1β*	NM_204524	F-CAGCCTCAGCGAAGAGACCTT	84	Xing et al. (15)
		R-ACTGTGGTGTGCTCAGAATCC		
*IL-6*	HM179640	F-AAATCCCTCCTCGCCAATCT	106	Xing et al. (15)
		R-CCCTCACGGTCTTCTCCATAAA		
*PPARγ*	NM_001001460.1	F-TCGCATCCATAAGAAAAGCA	176	Zhang et al. (41)
		R-CTTCTCCTTCTCCGCTTCGT		
*β-actin*	NM_205518	F-GCCAACAGAGAGAAGATGACAC	118	Xing et al. (15)
		R-GTAACACCATCACCAGAGTCCA		

TLR4, toll like receptor 4; MyD88, myeloid differentiation factor 88; TRAF6, tumor necrosis factor receptor associated factor 6; NF-κB p65, nuclear factor kappa B p65; NF-κB p50, nuclear factor kappa B p50; IκBα, inhibitor of NF-κB alpha; IL-1β, interleukin 1 beta; IL-6, interleukin 6; PPARγ, peroxisome proliferator-activated receptor γ; β-actin, beta-actin; F, forward primer; R, reverse primer.

### Statistical analysis

Data obtained are expressed as the mean with standard deviation (SD). The analyses of all data were performed by one-way ANOVA using GLM procedure of SAS 9.2 (SAS Institute Inc., Cary, NC, USA) with replicate as the experimental unit. Tukey's multiple range test was used to compare the mean values (*P* < 0.05) to show the significant differences.

## Results

### Growth performance

As shown in Table 3, dietary AOAE showed no significant effect on growth performance in broilers before LPS challenge (days 1 to 15) (*P* > 0.05). Compared with the control group, the broilers with LPS intraperitoneal injection alone presented a significant decrease in ADG and ADFI during the stress period and convalescence (*P* < 0.05). In contrast, the group that was supplemented with 1,000 mg/kg AOAE exhibited significant increase in ADG and ADFI (*P* < 0.05) compared with the LPS group, and had no significant difference compared to the control group (*P* > 0.05). Moreover, in the convalescence (days 29 to 42), broiler ADG in the AOAE-M group (LPS-challenged broilers fed a basal diet supplemented with 750 mg/kg AOAE) was higher than that in the LPS group (*P* < 0.05). Over the whole experimental period, there was no significant difference in the feed-to-gain ratio among broilers fed with AOAE or LPS-challenged (*P* > 0.05).

**Table 3:** Effect of AOAE on growth performance of broilers challenged with LPS.

**Items**	**Treatments**					***P*-value**
	**CON**	**LPS**	**AOAE-L**	**AOAE-M**	**AOAE-H**	
Starter phase (days 1 to 15)						
BW at day 1, g	38.07 ± 0.36	38.21 ± 0.49	38.17 ± 0.31	38.10 ± 0.46	38.18 ± 0.23	0.9672
ADG, g/d	26.45 ± 2.54	26.01 ± 2.60	27.12 ± 2.15	27.83 ± 1.48	28.50 ± 1.05	0.2514
ADFI, g/d	36.08 ± 2.98	36.37 ± 2.32	35.24 ± 3.93	36.99 ± 2.44	37.91 ± 1.22	0.5317
F: G	1.37 ± 0.03	1.40 ± 0.06	1.30 ± 0.14	1.33 ± 0.03	1.33 ± 0.06	0.2193
Stress period (days 16 to 28)						
BW at day 15, g	434.81 ± 38.03	428.33 ± 38.74	444.91 ± 32.11	455.48 ± 22.03	465.62 ± 15.90	0.2464
ADG, g/d	58.35 ± 3.96^a^	49.22 ± 3.69^c^	52.18 ± 5.20^bc^	52.16 ± 4.82^bc^	56.00 ± 4.12^ab^	0.0120
ADFI, g/d	92.89 ± 7.34^a^	80.30 ± 3.27^c^	81.91 ± 9.36^bc^	84.45 ± 7.03^abc^	90.13 ± 9.45^ab^	0.0378
F: G	1.60 ± 0.15	1.64 ± 0.14	1.58 ± 0.25	1.63 ± 0.14	1.61 ± 0.13	0.9803
Convalescence (days 29 to 42)						
ADG, g/d	85.73 ± 8.58^ab^	73.17 ± 6.51^c^	79.19 ± 5.15^bc^	84.34 ± 9.43^ab^	89.91 ± 5.56^a^	0.0054
ADFI, g/d	160.37 ± 11.49^a^	142.97 ± 11.82^b^	155.55 ± 9.11^ab^	155.05 ± 14.14^ab^	164.90 ± 6.93^a^	0.0252
F: G	1.88 ± 0.15	1.96 ± 0.14	1.97 ± 0.16	1.84 ± 0.11	1.84 ± 0.15	0.3613

1) BW, body weight; ADG, average daily gain; ADFI, average daily feed intake; F: G, feed-to-gain ratio.

2) AOAE, Artemisia ordosica alcohol extract; CON, non-challenged broilers fed a basal diet; LPS, lipopolysaccharide-challenged broilers fed a basal diet; AOAE-L, lipopolysaccharide-challenged broilers fed a basal diet supplemented with 500 mg/kg AOAE; AOAE-M, lipopolysaccharide-challenged broilers fed a basal diet supplemented with 750 mg/kg AOAE; AOAE-H, lipopolysaccharide-challenged broilers fed a basal diet supplemented with 1,000 mg/kg AOAE.

3) Each value is shown as mean ± SD (*n* = 6); Different superscript letters (a, b and c) within the same row indicate significant difference between experimental groups (*P* <0.05).

### Immune organ index

The effects of AOAE on the relative weight of immune organs in broilers challenged with LPS are shown in Table 4. As described in Table 4, on day 28, the spleen index markedly increased while the bursa of Fabricius index markedly decreased in the LPS group relative to broilers in the CON group, whereas this negative effect was obviously alleviated by feeding 750 and 1,000 mg/kg AOAE (*P* < 0.05). Likewise, compared with the control group, LPS caused increase of spleen index which was significantly inhibited by the treatments with AOAE at both 750 and 1,000 mg/kg on day 42 (*P* < 0.05). Broilers in AOAE-M and AOAE-H group (LPS-challenged broilers fed a basal diet supplemented with 1,000 mg/kg AOAE) had comparable relative weight of spleen (d 28 and 42) to broilers in the CON group (*P* > 0.05). There was no obvious difference in thymus index between LPS- and/or AOAE-treated broilers over the whole experimental period (*P* > 0.05).

**Table 4:** Effect of AOAE on relative weight of immune organs in broilers challenged with LPS.

**Items**	**Treatments**					***P*-value**
	**CON**	**LPS**	**AOAE-L**	**AOAE-M**	**AOAE-H**	
**28 d**						
Spleen index	0.93 ± 0.13^c^	1.60 ± 0.21^a^	1.39 ± 0.22^b^	1.09 ± 0.16^c^	0.96 ± 0.13^c^	<0.0001
Thymus index	2.19 ± 0.33	2.52 ± 0.39	2.33 ± 0.30	2.29 ± 0.30	2.27 ± 0.26	0.4965
Bursa index	2.67 ± 0.37^a^	1.73 ± 0.30^c^	1.86 ± 0.13^c^	2.25 ± 0.26^b^	2.60 ± 0.45^ab^	<0.0001
**42 d**						
Spleen index	1.07 ± 0.12^b^	1.28 ± 0.13^a^	1.18 ± 0.12^ab^	1.11 ± 0.11^b^	1.05 ± 0.11^b^	0.0135
Thymus index	2.42 ± 0.44	2.39 ± 0.41	2.44 ± 0.30	2.39 ± 0.39	2.38 ± 0.31	0.9983
Bursa index	1.81 ± 0.21	1.80 ± 0.16	1.83 ± 0.17	1.84 ± 0.27	1.83 ± 0.25	0.9973

1) Index = organ weight (g)/body weight (kg).

2) AOAE, Artemisia ordosica alcohol extract; CON, non-challenged broilers fed a basal diet; LPS, lipopolysaccharide-challenged broilers fed a basal diet; AOAE-L, lipopolysaccharide-challenged broilers fed a basal diet supplemented with 500 mg/kg AOAE; AOAE-M, lipopolysaccharide-challenged broilers fed a basal diet supplemented with 750 mg/kg AOAE; AOAE-H, lipopolysaccharide-challenged broilers fed a basal diet supplemented with 1,000 mg/kg AOAE.

3) Each value is shown as mean ± SD (n = 6); Different superscript letters (a, b, and c) within the same row indicate significant difference between experimental groups (*P* < 0.05).

### Effects of AOAE on serum cytokines and immunoglobulins in broilers challenged with LPS

As indicated in Table 5, on day 28, compared with the CON group, LPS challenge decreased the contents of IgM and IL-4 and increased the contents of IL-1β, IL-2 and IL-6 in serum of broilers; however, dietary AOAE supplementation obviously alleviated the decrease of IgM and IL-4 and the increase of IL-1β, IL-2 and IL-6 in the serum of broilers challenged with LPS, and the AOAE-H group had the best alleviatory effect (*P* < 0.05).

**Table 5:** Effect of AOAE on serum cytokines and immunoglobulins in broilers challenged with LPS.

**Items**	**Treatments**					***P*-value**
	**CON**	**LPS**	**AOAE-L**	**AOAE-M**	**AOAE-H**	
**28 d**						
IL-1β, pg/mL	53.10 ± 3.85^c^	69.57 ± 1.86^a^	64.19 ± 3.51^ab^	58.75 ± 6.72^bc^	55.39 ± 5.82^c^	<0.0001
IL-2, pg/mL	37.22 ± 2.07^c^	45.36 ± 5.98^a^	42.80 ± 2.83^ab^	39.30 ± 1.41^bc^	38.97 ± 5.25^bc^	0.0100
IL-4, pg/mL	22.95 ± 3.20^a^	17.88 ± 2.93^c^	19.04 ± 1.17^bc^	20.68 ± 2.37^abc^	21.06 ± 1.33^ab^	0.0104
IL-6, pg/mL	5.05 ± 0.16^c^	9.97 ± 0.36^a^	8.85 ± 0.30^b^	5.41 ± 0.57^c^	5.37 ± 0.60^c^	<0.0001
IgA, μg/mL	35.57 ± 2.75	36.64 ± 1.80	36.91 ± 2.89	35.34 ± 4.01	35.85 ± 2.62	0.8552
IgG, μg/mL	258.45 ± 17.01	290.14 ± 28.03	284.71 ± 25.42	260.55 ± 45.62	262.79 ± 33.85	0.2807
IgM, μg/mL	130.73 ± 12.70^a^	107.10 ± 4.86^c^	115.30 ± 7.50^bc^	125.13 ± 9.75^ab^	126.65 ± 4.47^a^	0.0003
**42 d**						
IL-1β, pg/mL	54.77 ± 8.45	59.94 ± 7.91	60.06 ± 9.51	60.05 ± 2.47	58.01 ± 6.13	0.6740
IL-2, pg/mL	36.40 ± 4.49	37.42 ± 3.95	35.61 ± 3.47	37.29 ± 2.62	36.38 ± 5.33	0.9377
IL-4, pg/mL	20.57 ± 3.00	19.51 ± 1.82	20.14 ± 2.13	19.07 ± 2.70	19.00 ± 1.98	0.7362
IL-6, pg/mL	5.01 ± 0.31	5.22 ± 0.65	5.13 ± 0.44	5.36 ± 0.25	5.52 ± 0.26	0.2495
IgA, μg/mL	32.29 ± 3.64	34.56 ± 5.11	31.29 ± 7.46	32.71 ± 5.25	31.23 ± 4.67	0.8190
IgG, μg/mL	314.14 ± 15.04	283.96 ± 10.68	283.19 ± 49.98	299.14 ± 39.19	292.13 ± 48.08	0.5792
IgM, μg/mL	150.55 ± 22.80	142.67 ± 8.93	142.24 ± 9.99	146.08 ± 12.90	155.11 ± 18.61	0.5719

1) IL-1β, interleukin 1 beta; IL-2, interleukin 2; IL-4, interleukin 4; IL-6, interleukin 6; IgA, immunoglobulin A; IgG, immunoglobulin G; IgM, immunoglobulin M.

2) AOAE, Artemisia ordosica alcohol extract; CON, non-challenged broilers fed a basal diet; LPS, lipopolysaccharide-challenged broilers fed a basal diet; AOAE-L, lipopolysaccharide-challenged broilers fed a basal diet supplemented with 500 mg/kg AOAE; AOAE-M, lipopolysaccharide-challenged broilers fed a basal diet supplemented with 750 mg/kg AOAE; AOAE-H, lipopolysaccharide-challenged broilers fed a basal diet supplemented with 1000 mg/kg AOAE.

3) Each value is shown as mean ± SD (n = 6); Different superscript letters (a, b, and c) within the same row indicate significant difference between experimental groups (*P* < 0.05).

The effect of AOAE addition and LPS challenge on serum cytokines and immunoglobulins in broilers during convalescence is also presented in Table 5. Neither the AOAE addition nor the LPS challenge had any effect on serum immunoglobulins and cytokines of broilers on day 42 (*P* > 0.05).

### Effects of AOAE and LPS treatment on the hepatic cytokines and immunoglobulins in broilers

As illustrated in Table 6, on day 28, stimulation with LPS alone caused a noticeable increase of IL-1β and IL-2 production in liver compared with the control group, whereas AOAE addition dramatically suppressed the overproduction of hepatic IL-1β (500, 750 and 1,000 mg/kg AOAE) and IL-2 (1,000 mg/kg AOAE) in LPS-treated broilers (*P* < 0.05). In addition, the hepatic IgM content in the AOAE-M and AOAE-H groups was significantly higher than that in the LPS group (*P* < 0.05). On day 42, the hepatic IL-2 content in AOAE-H group was lower than that in LPS group (*P* < 0.05), but had no difference from the CON group (*P* > 0.05).

**Table 6:** Effect of AOAE on hepatic cytokines and immunoglobulins in broilers challenged with LPS.

**Items**	**Treatments**					***P*-value**
	**CON**	**LPS**	**AOAE-L**	**AOAE-M**	**AOAE-H**	
**28 d**						
IL-1β, pg/mg prot.	9.4 ± 1.28^c^	31.98 ± 1.69^a^	23.42 ± 1.44^b^	9.96 ± 0.48^c^	9.45 ± 1.12^c^	<0.0001
IL-2, pg/mg prot.	4.19 ± 0.27^c^	9.86 ± 0.48^a^	9.28 ± 0.43^ab^	9.03 ± 1.06^ab^	8.61 ± 1.54^b^	<0.0001
IL-4, pg/mg prot.	2.33 ± 0.16	2.44 ± 0.25	2.51 ± 0.12	2.58 ± 0.24	2.37 ± 0.27	0.2689
IL-6, pg/mg prot.	0.51 ± 0.02	0.54 ± 0.04	0.52 ± 0.04	0.53 ± 0.07	0.49 ± 0.07	0.4774
IgA, μg/mg prot.	6.54 ± 0.23	5.94 ± 0.54	6.49 ± 0.56	6.80 ± 0.62	6.66 ± 0.92	0.1845
IgG, μg/mg prot.	47.99 ± 3.56	48.56 ± 2.79	48.34 ± 4.94	48.95 ± 3.96	48.26 ± 7.88	0.9979
IgM, μg/mg prot.	13.08 ± 1.48^bc^	11.35 ± 0.95^c^	13.25 ± 1.63^abc^	13.52 ± 0.94^ab^	15.11 ± 2.33^a^	0.0075
**42 d**						
IL-1β, pg/mg prot.	13.04 ± 2.02	15.06 ± 2.58	14.69 ± 2.24	14.37 ± 2.70	12.63 ± 2.38	0.3447
IL-2, pg/mg prot.	6.01 ± 0.80^ab^	6.76 ± 0.55^a^	6.76 ± 1.24^a^	6.81 ± 0.96^a^	5.27 ± 0.83^b^	0.0244
IL-4, pg/mg prot.	3.26 ± 0.67	3.49 ± 0.42	3.82 ± 0.93	3.55 ± 0.61	3.03 ± 0.56	0.3227
IL-6, pg/mg prot.	0.66 ± 0.11	0.70 ± 0.04	0.73 ± 0.16	0.77 ± 0.11	0.62 ± 0.16	0.2713
IgA, μg/mg prot.	9.68 ± 0.57	8.15 ± 0.93	8.39 ± 2.21	10.06 ± 1.28	10.19 ± 2.70	0.1569
IgG, μg/mg prot.	73.01 ± 1.39	70.18 ± 5.79	77.42 ± 19.14	71.77 ± 10.17	72.82 ± 18.60	0.9031
IgM, μg/mg prot.	20.75 ± 2.26	18.25 ± 2.98	20.10 ± 3.87	20.37 ± 2.82	20.10 ± 6.36	0.8329

1) IL-1β, interleukin 1 beta; IL-2, interleukin 2; IL-4, interleukin 4; IL-6, interleukin 6; IgA, immunoglobulin A; IgG, immunoglobulin G; IgM, immunoglobulin M.

2) AOAE, Artemisia ordosica alcohol extract; CON, non-challenged broilers fed a basal diet; LPS, lipopolysaccharide-challenged broilers fed a basal diet; AOAE-L, lipopolysaccharide-challenged broilers fed a basal diet supplemented with 500 mg/kg AOAE; AOAE-M, lipopolysaccharide-challenged broilers fed a basal diet supplemented with 750 mg/kg AOAE; AOAE-H, lipopolysaccharide-challenged broilers fed a basal diet supplemented with 1,000 mg/kg AOAE.

3) Each value is shown as mean ± SD (n = 6); Different superscript letters (a, b, and c) within the same row indicate significant difference between experimental groups (*P* < 0.05).

### AOAE regulated the expression of TLR4/NF-κB pathway related genes in liver of broilers challenged with LPS

**Figure 1:**
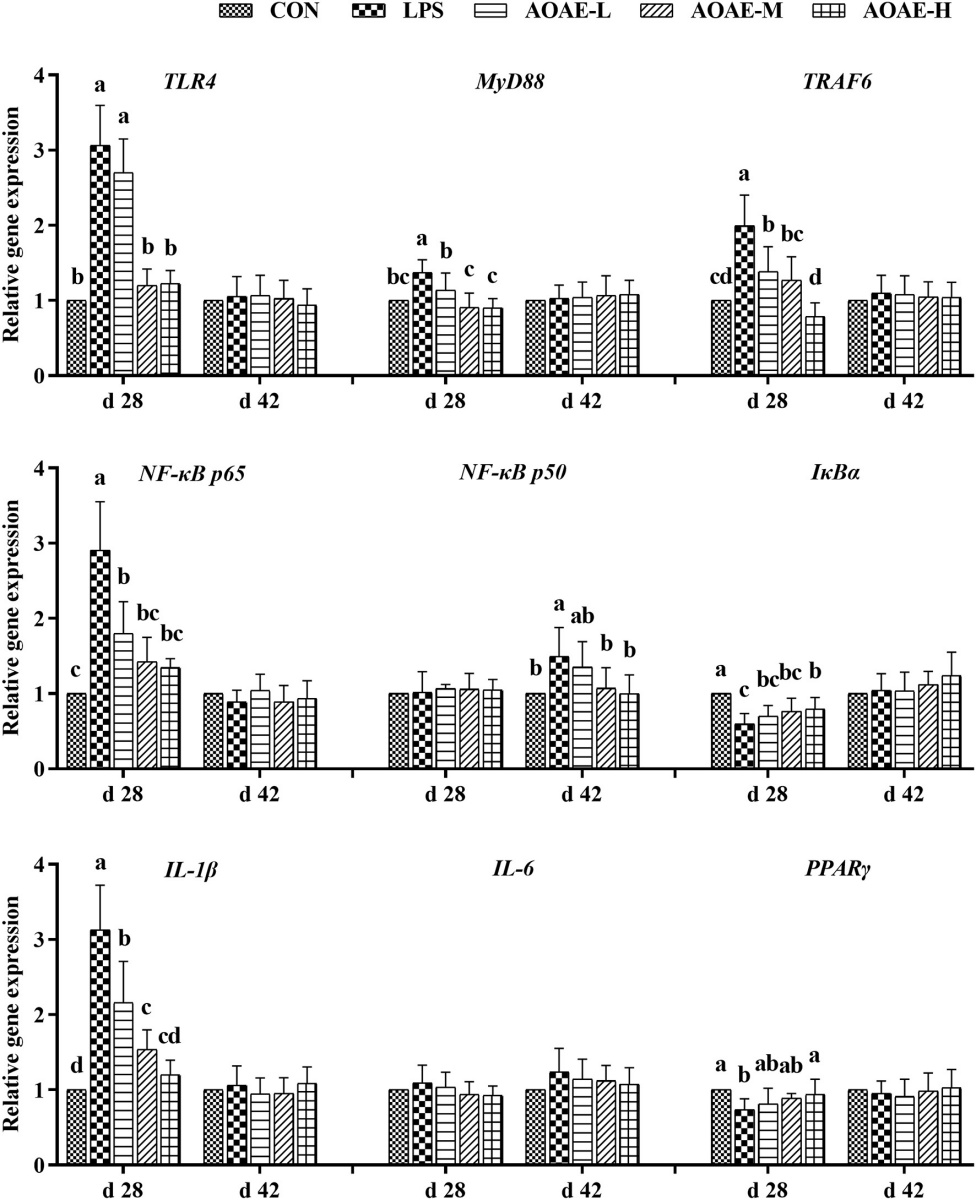
Effect of AOAE on the expression of TLR4/NF-κB pathway related genes in liver of broilers challenged with LPS (1) TLR4, toll like receptor 4; MyD88, myeloid differentiation factor 88; TRAF6, tumor necrosis factor receptor associated factor 6; NF-κB p65, nuclear factor kappa B p65; NF-κB p50, nuclear factor kappa B p50; IκBα, inhibitor of NF-κB alpha; IL-1β, interleukin 1 beta; IL-6, interleukin 6; PPARγ, peroxisome proliferator-activated receptor γ. (2) AOAE, *Artemisia ordosica* alcohol extract; CON, non-challenged broilers fed a basal diet; LPS, lipopolysaccharide-challenged broilers fed a basal diet; AOAE-L, lipopolysaccharide-challenged broilers fed a basal diet supplemented with 500 mg/kg AOAE; AOAE-M, lipopolysaccharide-challenged broilers fed a basal diet supplemented with 750 mg/kg AOAE; AOAE-H, lipopolysaccharide-challenged broilers fed a basal diet supplemented with 1,000 mg/kg AOAE. (3) The gene expression for β*-actin* was used as a housekeeping gene. The relative expression levels from the control group were used as reference values. Each value is shown as mean ± SD (n = 6). Bars that do not share the same letters (a, b, c, and d) are significantly different (*P* < 0.05) from each other.

To determine whether the anti-inflammatory action of AOAE was mediated through the TLR4/NF-κB signaling pathway, the gene expression of *TLR4, MyD88, TRAF6, NF-*κ*B p65, NF-*κ*B p50, I*κ*B*α, *IL-1*β, *IL-6*, and *PPAR*γ was assessed by quantitative real-time PCR analysis. As summarized in Figure 1, on day 28, compared to the control group, LPS administration increased the gene expression of *TLR4, MyD88, TRAF6, NF-*κ*B p65*, and *IL-1*β, but decreased the gene expression of *I*κ*B*α and *PPAR*γ in the liver tissue of broilers (*P* < 0.05); however, the negative changes in mRNA expression of the aforementioned genes were markedly reversed by adding AOAE in diets, except for *TLR4*, which decreased significantly only with 750 and 1,000 mg/kg AOAE supplementation (*P* < 0.05). On day 42, hepatic *NF-*κ*B p50* mRNA expression in AOAE-M and AOAE-H groups was lower than that in the LPS group (*P* < 0.05), but had no difference from the CON group (*P* > 0.05).

### Effects of AOAE and LPS treatment on the splenic cytokines and immunoglobulins in broilers

As illustrated in Table 7, on day 28, the broilers that were injected with LPS only exhibited a marked increase of the IL-1β and IL-6 in spleen compared with the control group, whereas AOAE addition remarkably reduced the upregulated levels of IL-1β (500, 750, and 1,000 mg/kg AOAE) and IL-6 (1,000 mg/kg AOAE) (*P* < 0.05). Similarly, the contents of splenic IgG and IgM were significantly decreased under LPS challenge, while AOAE supplementation markedly reversed the downregulated levels of IgG (1,000 mg/kg AOAE) and IgM (500, 750, and 1,000 mg/kg AOAE) (*P* < 0.05). On day 42, the splenic IgG and IgM contents in AOAE-H group were higher than that in the LPS group (*P* < 0.05), but had no difference from the CON group (*P* > 0.05). In addition, the IL-1β level in LPS group was significantly higher than that in the other groups during convalescence (*P* < 0.05).

**Table 7:** Effect of AOAE on splenic cytokines and immunoglobulins in broilers challenged with LPS.

**Items**	**Treatments**					***P*-value**
	**CON**	**LPS**	**AOAE-L**	**AOAE-M**	**AOAE-H**	
**28 d**						
IL-1β, pg/mg prot.	13.09 ± 2.19^c^	33.32 ± 3.77^a^	26.19 ± 2.33^b^	23.23 ± 3.49^b^	14.73 ± 2.32^c^	<0.0001
IL-2, pg/mg prot.	6.40 ± 0.97	8.06 ± 0.92	7.75 ± 1.31	7.59 ± 1.44	7.33 ± 1.57	0.2354
IL-4, pg/mg prot.	4.01 ± 0.59	3.93 ± 0.57	4.56 ± 0.76	4.48 ± 0.75	4.51 ± 0.52	0.2928
IL-6, pg/mg prot.	0.59 ± 0.11^c^	2.71 ± 0.49^a^	2.75 ± 0.37^a^	2.70 ± 0.18^a^	0.97 ± 0.17^b^	<0.0001
IgA, μg/mg prot.	9.13 ± 1.09	8.22 ± 0.74	8.70 ± 1.39	9.11 ± 1.52	8.78 ± 0.47	0.6152
IgG, μg/mg prot.	84.32 ± 10.57^a^	65.68 ± 9.61^b^	60.18 ± 11.25^b^	63.03 ± 11.32^b^	87.41 ± 16.79^a^	0.0009
IgM, μg/mg prot.	27.78 ± 1.86^a^	16.93 ± 1.96^c^	22.00 ± 1.79^b^	26.33 ± 5.30^a^	26.71 ± 3.71^a^	<0.0001
**42 d**						
IL-1β, pg/mg prot.	11.36 ± 1.30^b^	15.27 ± 3.13^a^	12.17 ± 2.16^b^	11.47 ± 1.69^b^	12.55 ± 1.65^b^	0.0214
IL-2, pg/mg prot.	8.60 ± 0.85	7.86 ± 1.65	8.00 ± 1.19	7.76 ± 1.17	7.85 ± 1.81	0.8300
IL-4, pg/mg prot.	4.12 ± 0.47	3.92 ± 0.72	3.66 ± 0.58	4.27 ± 0.59	4.54 ± 0.91	0.2318
IL-6, pg/mg prot.	0.70 ± 0.09	0.71 ± 0.11	0.73 ± 0.06	0.69 ± 0.10	0.71 ± 0.10	0.9742
IgA, μg/mg prot.	6.96 ± 1.13	7.35 ± 0.72	6.85 ± 1.10	6.73 ± 0.97	7.56 ± 1.50	0.6689
IgG, μg/mg prot.	72.84 ± 4.72^ab^	64.50 ± 7.08^b^	66.13 ± 9.40^b^	73.13 ± 7.98^ab^	83.72 ± 16.62^a^	0.0224
IgM, μg/mg prot.	27.26 ± 4.97^a^	19.14 ± 2.86^c^	21.23 ± 3.60^bc^	22.60 ± 3.27^bc^	24.35 ± 3.14^ab^	0.0087

1) IL-1β, interleukin 1 beta; IL-2, interleukin 2; IL-4, interleukin 4; IL-6, interleukin 6; IgA, immunoglobulin A; IgG, immunoglobulin G; IgM, immunoglobulin M.

2) AOAE, Artemisia ordosica alcohol extract; CON, non-challenged broilers fed a basal diet; LPS, lipopolysaccharide-challenged broilers fed a basal diet; AOAE-L, lipopolysaccharide-challenged broilers fed a basal diet supplemented with 500 mg/kg AOAE; AOAE-M, lipopolysaccharide-challenged broilers fed a basal diet supplemented with 750 mg/kg AOAE; AOAE-H, lipopolysaccharide-challenged broilers fed a basal diet supplemented with 1000 mg/kg AOAE.

3) Each value is shown as mean ± SD (n = 6); Different superscript letters (a, b, and c) within the same row indicate significant difference between experimental groups (*P* <0.05).

### AOAE regulated the expression of TLR4/NF-κB pathway related genes in spleen of broilers challenged with LPS

**Figure 2:**
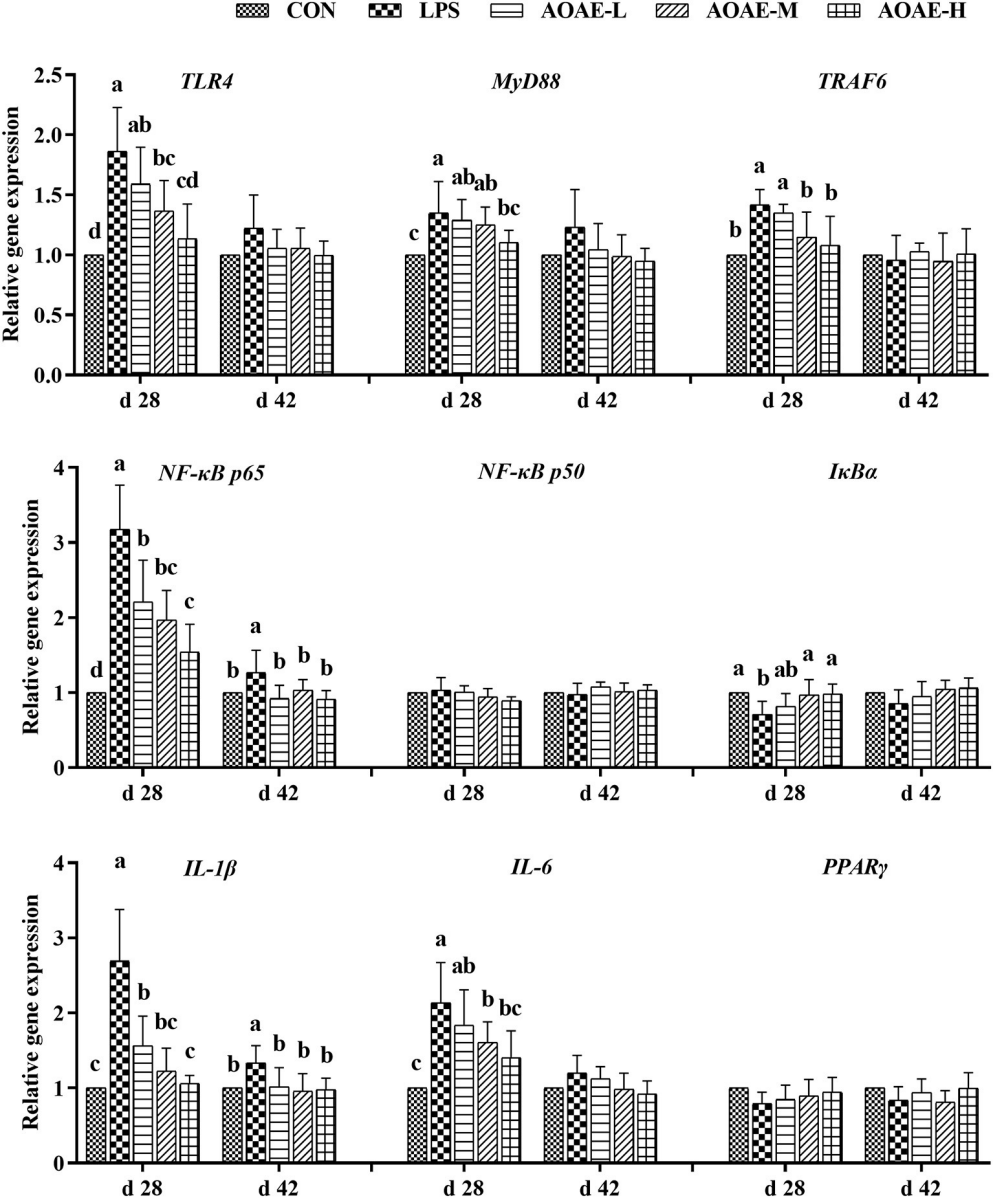
Effect of AOAE on the expression of TLR4/NF-κB pathway related genes in spleen of broilers challenged with LPS. (1) TLR4, toll like receptor 4; MyD88, myeloid differentiation factor 88; TRAF6, tumor necrosis factor receptor associated factor 6; NF-κB p65, nuclear factor kappa B p65; NF-κB p50, nuclear factor kappa B p50; IκBα, inhibitor of NF-κB alpha; IL-1β, interleukin 1 beta; IL-6, interleukin 6; PPARγ, peroxisome proliferator-activated receptor γ. (2) AOAE, Artemisia ordosica alcohol extract; CON, non-challenged broilers fed a basal diet; LPS, lipopolysaccharide-challenged broilers fed a basal diet; AOAE-L, lipopolysaccharide-challenged broilers fed a basal diet supplemented with 500 mg/kg AOAE; AOAE-M, lipopolysaccharide-challenged broilers fed a basal diet supplemented with 750 mg/kg AOAE; AOAE-H, lipopolysaccharide-challenged broilers fed a basal diet supplemented with 1000 mg/kg AOAE. (3) The gene expression for β-actin was used as a housekeeping gene. The relative expression levels from the control group were used as reference values. Each value is shown as mean ± SD (*n* = 6). Bars that do not share the same letters (a, b, c and d) are significantly different (*P* <0.05) from each other.

The splenic gene expression data from the broilers are shown in Figure 2. On day 28, LPS markedly elevated the gene expression of *TLR4, MyD88, TRAF6, NF-*κ*B p65, IL-1*β, and *IL-6*, whereas decreased the gene expression of *I*κ*B*α in the spleen tissue of broilers (*P* <0.05); however, dietary AOAE supplementation obviously alleviated negative changes induced by LPS, and the AOAE-H group had the best alleviatory effect (*P* <0.05). On day 42, the splenic *NF-*κ*B p65* and *IL-1*β mRNA expression in LPS group were significantly higher than those in the other groups (*P* <0.05).

## Discussions

In this experiment, we used a classical LPS-stimulated model to induce the inflammation in broilers by intraperitoneal administration of LPS and evaluated the effect of AOAE on LPS induced inflammatory immune response broiler model. As observed in the present investigation, *E. coli* LPS injection caused the inflammatory reaction and obviously inhibited the growth performance of broilers, which suggested that the LPS simulation model had been successfully established, consistent with our previous study (26, 42).

In the current study, we found that LPS challenge (LPS group) decreased the ADG and ADFI compared with that in the unchallenged broilers (CON group), which was consistent with the findings of Li et al. (26) and Yang et al. (42), where LPS-induced growth retardation was reported in broilers exposed to 500 μg/kg BW LPS. This phenomenon might be partly due to dietary nutrients being diverted from maintaining growth to maintaining the stability of the immune system, and partly to the excessive production of inflammatory cytokines (IL-1β and IL-6) under inflammation feed information back to the central nervous system to suppress the animal's appetite. Simultaneously, we also observed that, diet supplemented with 1,000 mg/kg of AOAE could improve the ADG and ADFI in broilers stimulated by LPS both in stress period and convalescence, thereby alleviating the inhibition of the growth performance of broilers caused by LPS challenge, which was consistent with the previous studies (26, 42). This may be related to the fact that AOAE contained flavonoids and polysaccharides that were able to alleviate LPS-induced inflammation by suppressing the release of proinflammatory cytokines, ultimately improving the growth performance. Furthermore, Xing et al. (27) reported that flavonoids and polysaccharides of *A. ordosica* aqueous extract might contribute to the positive effects on growth performance by improving the apparent nutrient digestibility of weanling piglets in a dose-dependent manner. Another study indicated that LPS and *A. ordosica* extract (rich in flavonoids and phenolic acids) exhibited an interaction for the content of insulin-like growth factor-1 (IGF-1) in serum; in other words, *A. ordosica* extract (rich in flavonoids and phenolic acids) could also relieve the growth inhibition of broilers challenged with LPS by promoting the secretion of growth-promoting hormones (26). More specifically, it has been proven that flavone can promote the combination of growth hormone and hepatic growth hormone receptor and then induce growth promotion (43). These results may also partially explain our findings.

The thymus, bursa of Fabricius, and spleen are important immune organs in birds, which all play a key function in defending the body against external or internal irritants or pathogens through different immune response mechanisms. B-lymphocytes mature in the bursa of Fabricius, while the thymus is responsible for the differentiation and proliferation of T-lymphocytes (44, 45). As the largest peripheral immune organ and an initiating site for antibody production after antigenic stimulation, the spleen can regulate cellular and humoral immunity in poultry via activation of B-lymphocytes, T-lymphocytes, and macrophages (46). Hence, their relative development is indispensable in evaluating the poultry immune system and health condition. Consistent with the results of Ahiwe et al. (47), in the stress period of the current study, compared with the CON group, an increase in spleen index and a decline in bursa index were found in the broilers of LPS group after LPS challenge. The increase in the relative weight of spleen observed in LPS group may be associated with the compensatory splenic hyperplasia induced by LPS, which resulted in the activation of inflammatory cells and the large-scale production of inflammatory cytokines (48). Furthermore, as the most responsive immune organ of broiler for microbial infections, in the stress period of the present study, compared with the CON group, the bursa was observed a decrease in the relative weight in response to LPS challenge, which may be due to the occurrence of bursa immune overresponse caused by the presence of LPS antigen that disrupted histo-morphological integrity, as well as pro-apoptotic factors that induce apoptosis and chronic atrophy of the bursa (47). The increase of spleen index and decrease of bursa index in LPS-challenged broilers were noticeably alleviated after dietary supplementation with 1,000 mg/kg AOAE, which demonstrated that AOAE might be able to have beneficial effects on homeostatic mechanisms associated with the immune response of immune organs under inflammatory conditions. These results were in agreement with a previous study of flavonoid-rich ethanolic extract, which reported a significant improvement in heart, thymus and bursa size of coccidia-challenged broilers (49). The reason for this improvement induced by AOAE supplementation in diets of broilers challenged with LPS might be associated with the ability of flavonoids to optimize immunity by promoting the phagocytic activity of macrophages, therefore, alleviating the damage of visceral organs caused by pathogenic bacteria and viruses (50).

The immune protective properties and immunomodulatory potential of flavonoids from medicinal plants in living systems have been reported. Zhong et al. (13) also found that flavonoids (3,5,3′,4′-tetrahydroxy-6,7-dimethoxyflavone, 5,3′,4′-trihydroxy-7-methoxyflavanone, 5,7,4′-trihydroxy-6-methoxyflavone) isolated from *A. ordosica* exhibited strong inhibitory activities for the formation of nitric oxide (NO) in rat macrophages *in vitro*. Unfortunately, to our knowledge, little or no previous study has attempted to examine the *in vivo* alleviating effects of AOAE on LPS induced inflammatory reaction. The present study found that, compared with the CON group, intraperitoneal injection with 750 μg/kg BW LPS caused overproduction of IL-1β, IL-2, and IL-6 in the serum of broilers in LPS group during stress period, which could prove the successful induction of inflammatory reaction in broilers. Meanwhile, we observed a decreased IL-4 and IgM level in serum after LPS challenge in LPS group broilers, indicating that LPS challenge disturbed the balance of the proinflammatory and anti-inflammatory systems. However, dietary supplementation of both 750 and 1,000 mg/kg AOAE were able to decrease the level of these cytokines and increase the content of IL-4 and IgM in broilers challenged by LPS, which could protect against LPS-induced inflammatory responses. These benefits were possibly due to the immunomodulatory characteristic of AOAE, mainly provided by flavonoids. This hypothesis was also supported by the previous findings of Niu et al. (51), who reported that naringenin from *A. ordosica* could increase the level of the anti-inflammatory factor IL-4, while decrease the inflammatory factor tumor necrosis factor α (TNF-α). Similarly, another study reported that *Artemisia anomala* ethanolic extract, the most potent ingredients of which were flavonoid compounds, had anti-inflammatory effects by inhibiting nod-like receptor family pyrin domain containing 3 (NLRP3) inflammasome activation (52). Combined with these results and based on our study, it is reasonable to infer that AOAE, whose main active ingredient is flavonoid, can inhibit LPS-induced proinflammatory responses to alleviate inflammation.

To gain further insight into the mitigating effects of AOAE against LPS-induced excessive inflammation in broilers, we selected the liver and spleen as the target organs. Undoubtedly, in addition to the spleen, the liver is also a major immune organ and plays a pivotal role in protein synthesis and detoxification processes, and has a high metabolic rate of nutrients from dietary feed (53). Many bioactive compounds, such as flavonoids and polysaccharides, can reach the liver and exert a variety of biological functions via the portal vein after absorption along the gastrointestinal tract (54, 55). As the target organ of LPS, the liver, while defending against bacteria and getting rid of their toxic products, such as LPS, is susceptible to damage from endotoxins and live bacteria (56). Immunoglobulins, as a vital part of the humoral immune system, are all produced by B lymphocytes and have many important biological functions, including recognizing and resisting pathogen invasion, enhancing immune ability, and neutralizing toxins, while also playing an important role in the regulation of inflammation (57). LPS stimulation has been confirmed to influence the expression level of immunoglobulins and inhibit the humoral immune response (58). Our results showed that intraperitoneal LPS injection led to a decrease in IgM and IgG in LPS group compared to the CON group, which was in line with previous studies. IgM is the first antibody generated during the primary antibody response post-infection and hence may be depleted or reduced to decrease or counter the antigen level when it encounters the antigens (59). IgG, also referred to as IgY in poultry, is the most abundant immunoglobulin and generated mainly in T-cell-dependent secondary antibody responses, which promotes immune cells to neutralize bacterial toxins (60, 61). In the current study, the LPS-induced decrease in IgM and IgG levels were manifestly moderated by the presence of AOAE with the level of 1,000 mg/kg, indicating the immune promotion effects of AOAE supplementation. Consistently, it was reported that supplementation with quercetin was able to ameliorate the decline in the levels of serum IgG, IgM, and IgE in rats exposed to doxorubicin (62). According to the results of this experiment, it is speculated that AOAE with flavonoids as the main active component can effectively alleviate the negative effects of inflammation caused by LPS stimulation via regulating the production of immunoglobulins, which help to resist further damage. Nevertheless, we observed that the immunoglobulin levels in the serum returned to normal, but the low levels in the spleen continued throughout the convalescence, suggesting that spleen recover more slowly after parenchymatous injury, whereas the recovery process could be accelerated by diet addition of 1,000 mg/kg AOAE.

Proinflammatory cytokines have a significant contribution in the initiation of inflammatory pathogenesis and the development of inflammatory pathology (63). In the present study, our results showed that, compared with the CON group, the LPS group had higher IL-1β and IL-2 in liver and IL-1β and IL-6 in spleen. However, we also found that hepatic IL-1β, IL-2, and splenic IL-1β, IL-6 of broilers challenged by LPS were lowered by 1,000 mg/kg AOAE supplementation during stress period, which indicated that AOAE exerted a certain degree of palliation for organ inflammation induced by LPS, and this effect was continuous, even in the convalescence after LPS stimulation ended. In addition, compared with the LPS group, the gene expression levels of hepatic and splenic IL-1β and splenic IL-6 in AOAE-M group and AOAE-H group were significantly decreased and restored to the levels of CON group in stress period and/or convalescence, which partially supports this finding. A similar result was reported for bovine mammary epithelial cells by Song et al. (64), who found that the ethanol extract of *Artemisia annua* (rich in flavonoids and phenolic acids) could exert anti-inflammatory effects by downregulating the mRNA levels of inflammatory cytokines, including TNF-α, IL-1β, and IL-6, in a dose-dependent manner. In our research, we found that AOAE may act as a protective agent and prevent LPS induced hepatic and splenic inflammatory injury to a certain extent, as evidenced by alleviating LPS-induced immune overresponse through suppressing the transcriptional expression and the secretion of inflammatory cytokines, which ultimately improved the systemic anti-inflammatory response.

Toll-like receptors (TLRs), acting as sentinels of pathogens, are a class of transmembrane proteins that play a crucial part in activating immunity and controlling inflammation, especially TLR4 (65). As the pattern recognition receptor of LPS, TLR4 mediates inflammatory responses via combining with MyD88 to activate various intracellular signaling pathways, including the NF-κB transduction cascades, upon LPS challenge (66). Normally, functional NF-κB dimers combine with its inhibitor protein IκB in the cytosol and act as an inactive form (67). When under inflammatory stimuli, NF-κB will dissociate from IκB, which is triggered by another kinase, IκB kinase (IKK), and subsequently transfer into the nucleus, where it upregulates the expression of proinflammatory cytokines, including IL-1β, IL-6, TNF-α, and IL-2, eventually exaggerates the initial inflammatory responses (67). Numerous studies have suggested that NF-κB, a crucial transcription factor for inflammation progression, is involved in the immunoregulation activity of flavonoids and polysaccharides isolated from a variety of plants (50, 61). As an extension, we speculated that AOAE has a similar anti-inflammatory effect against the inflammatory disorders caused by LPS-challenge and primarily explored the possible action mechanism by detecting the mRNA expressions of eight candidate key nodes in the TLR4/NF-κB signaling pathways in liver and spleen, including *TLR4, MyD88, TRAF6, NF-*κ*B p50, NF-*κ*B p65, I*κ*B*α, *IL-1*β, and *IL-6*. In the present study, the results showed that compared with the CON group, when broilers exposed to LPS (LPS group), the gene expression levels of *TLR4, MyD88, TRAF6, NF-*κ*B p65, IL-1*β, and *IL-6* were increased, while the gene expression level of *I*κ*B*α was decreased in liver and spleen. However, 1,000 mg/kg AOAE inhibited the gene over-expression of *NF-*κ*B p50* and *NF-*κ*B p65* through decreasing *TLR4, MyD88*, and *TRAF6* gene expression, but increasing *I*κ*B*α gene expression. Accordingly, as discussed above, the content of proinflammatory cytokines, such as IL-1β and IL-6, and the related gene expression were key to alleviate inflammation, and could be significantly reduced and restored to normal levels by AOAE. In addition, peroxisome proliferator activated-receptors (PPARs) have been shown to interact with NF-κB pathways and are believed to act as a class of anti-inflammatory proteins that have the ability to regulate inflammatory gene expression (68). Among them, PPARγ is known as a significant anti-inflammatory factor that can inhibit NF-κB by preventing its binding to target sequences (69). The data from the current study indicated that dietary AOAE supplementation for broilers alleviated the inhibitory effect of LPS on *PPAR*γ, which was similar to the finding of Lv et al. (70), who reported that genistein treatment upregulated the mRNA expressions of *PPAR*α in LPS-challenged chicks. It is therefore reasonable to assume that negative inflammation response regulators can also be activated by AOAE when the host is infected by pathogenic bacteria in a bid to moderate or restore immune homeostasis. The presence of flavonoids and polysaccharides in AOAE might be responsible for these benefits, as was previously described in broilers, in which the supplementation of *Artemisia argyi* flavonoids or *Artemisia ordosica* polysaccharides lowered serum inflammatory cytokines and improved immune function (21, 50). What we stress here is that the advantages of flavonoids should contribute indispensably to AOAE. Flavonoids isolated from *Artemisia ordosica* have been confirmed *in vitro* in both antioxidant and anti-inflammatory aspects (13, 20). Niu et al. (51) also found that the naringenin from *A. ordosica* could ameliorate the allergic rhinitis symptoms by increasing the IL-4 and decreasing the TNF-α, which showed an anti-allergic rhinitis effect. Additionally, as the main component in AOAE was total flavonoid (556.1 mg rutin/g AOAE), it is reasonable to speculate that AOAE may have the same anti-inflammatory effect as rutin which has been proven to ameliorate dextran sulfate sodium-induced colitis in mice by attenuating proinflammatory gene expression (71). The compounds that accounts for the anti-inflammatory effect of AOAE need to be clarified in the near future. Moreover, whether the anti-inflammatory effect of entire AOAE on LPS-exposed broilers is greater than that of the single active ingredient needs a continued study to confirm.

Actually, inflammation is generally not harmful to the pathogen and, in contrast, benefits the pathogen by providing an additional nutritional supply through the increased blood supply (72). The present results suggested the possibility that AOAE could suppress the LPS-mediated excessive production of inflammatory cytokines and recover the physiological balance. This might be related to the mediating of MyD88-dependent TLR4/NF-κB signaling pathway. As reported, the *Artemisia annua* ethanol extract (rich in flavonoids and phenolic acids) ameliorated inflammation by regulating NF-κB pathways (64). However, the effects of AOAE on the NF-κB signaling pathway still need to be validated by further studies. It is worth noting that the presence of different active constituents in herbal extract can simultaneously act on different targets in the inflammatory pathway (73). Increasing evidences demonstrate that the excess production of ROS is usually associated with high levels of proinflammatory cytokines, suggesting that crosstalk between the NF-κB and other pathways including nuclear factor erythroid 2-related factor 2 (Nrf2) may be an important regulatory mechanism in many cellular responses to various stresses, such as oxidative stress and immunological stress.

Collectively, *A. ordosica* has a great deal of advantages, such as abundant resources, low price, rich nutritional value and high pharmacological activity, and possesses great application and development value. Unfortunately, as far as we know, studies on the effects of *A. ordosica* and its extract as a feed additive on growth performance and health of livestock are limited, especially the study of *A. ordosica* alcohol extract in poultry. Consequently, further studies of different *in vitro* and *in vivo* disease models should be carried out to completely evaluate the role and the exact mechanism of AOAE in antioxidant and immunomodulatory.

## Conclusion

In conclusion, the present study reported for the first time that AOAE protected against LPS-induced inflammation in an animal model. The results obtained from this study showed that LPS injection inhibited growth performance of broilers and led to an inflammatory response manifested as high levels of IL-1β and IL-6 and low levels of IgG and IgM, which negatively affected several parameters including splenic and bursal relative weight. However, dietary AOAE supplementation could reverse these effects to improve growth performance of LPS-challenged broilers. Based on the results of our study, we preliminarily speculated that AOAE with flavonoids as the main active component could alleviate LPS-induced inflammatory response, which might be related to the mediating of TLR4/NF-κB pathways. Therefore, under the conditions of this experiment, the recommended level of AOAE in broiler diet was 1,000 mg/kg. AOAE deserves further investigation to develop a natural feed additive in poultry diets and improve growth performance and immune function in broilers.

## Data availability statement

The datasets presented in this study can be found in online repositories. The names of the repository/repositories and accession number(s) can be found in the article/supplementary material.

## Ethics statement

The animal study was reviewed and approved by the Animal Care and Use Committee of Inner Mongolia Agricultural University and performed following the national standard Guideline for Ethical Review of Animal Welfare (GB/T 35892-2018).

## Author contributions

Conceptualization and supervision: BS and SY. Investigation: LS. Sample collection: LS, YG, and YC. Data curation: LS and YC. Writing—original draft preparation: LS and LZ. Writing—review & editing: LS, YXi, and SG. Supervision: YXu and XJ. All authors contributed to the article and approved the submitted version.

## Conflict of interest

The authors declare that the research was conducted in the absence of any commercial or financial relationships that could be construed as a potential conflict of interest.

## Publisher's note

All claims expressed in this article are solely those of the authors and do not necessarily represent those of their affiliated organizations, or those of the publisher, the editors and the reviewers. Any product that may be evaluated in this article, or claim that may be made by its manufacturer, is not guaranteed or endorsed by the publisher.

## Abbreviations

LPS: lipopolysaccharide
ADG: average daily gain
ADFI: average daily feed intake
F, G: feed-to-gain ratio
IL: interleukin
Ig: immunoglobulin
TLR4: toll like receptor 4
MyD88: myeloid differentiation factor 88
TRAF6: tumor necrosis factor receptor associated factor 6
NF-κB p65: nuclear factor kappa B p65
NF-κB p50: nuclear factor kappa B p50
IκBα: inhibitor of NF-κB alpha
IL-1β: interleukin 1 beta
IL-6: interleukin 6
PPARγ: peroxisome proliferator-activated receptor γ.
